# Cardiovascular Outcomes in Patients on Home Hemodialysis and Peritoneal Dialysis

**DOI:** 10.34067/KID.0000000000000360

**Published:** 2024-02-01

**Authors:** Silvi Shah, Eric Weinhandl, Nupur Gupta, Anthony C. Leonard, Annette L. Christianson, Charuhas V. Thakar

**Affiliations:** 1 Division of Nephrology and Hypertension, Department of Internal Medicine, University of Cincinnati, Cincinnati, Ohio; 2 Satellite Healthcare, San Jose, California; 3 Department of Pharmaceutical Care and Health Systems, University of Minnesota, Minneapolis, Minnesota; 4 Division of Nephrology, Indiana University, Division of Nephrology, Indianapolis, Indiana; 5 Department of Environmental Health, University of Cincinnati, Cincinnati, Ohio; 6 Wellcome-Wolfson Institute for Experimental Medicine, School of Medicine, Dentistry and Biomedical Sciences, Queen's University Belfast, United Kingdom; 7 Division of Nephrology, VA Medical Center, Cincinnati, Ohio

**Keywords:** cardiovascular events, peritoneal dialysis, home hemodialysis, death, cardiovascular disease, hemodialysis, kidney failure

## Abstract

**Key Points:**

- Home hemodialysis is associated with decreased risk of stroke and acute coronary syndrome relative to peritoneal dialysis.
- Home hemodialysis is associated with decreased risk of cardiovascular death and all-cause death relative to peritoneal dialysis.

**Background:**

Cardiovascular disease is the leading cause of morbidity and mortality in patients with ESKD. Little is known about differences in cardiovascular outcomes between home hemodialysis (HHD) and peritoneal dialysis (PD).

**Methods:**

We evaluated 68,645 patients who initiated home dialysis between January 1, 2005, and December 31, 2018, using the United States Renal Data System with linked Medicare claims. Rates for incident cardiovascular events of acute coronary syndrome, heart failure, and stroke hospitalizations were determined. Using adjusted time-to-event models, the associations of type of home dialysis modality with the outcomes of incident cardiovascular events, cardiovascular death, and all-cause death were examined.

**Results:**

Mean age of patients in the study cohort was 64±15 years, and 42.3% were women. The mean time of follow-up was 1.8±1.6 years. The unadjusted cardiovascular event rate was 95.1 per thousand person-years (PTPY) (95% confidence interval [CI], 93.6 to 96.8), with a higher rate in patients on HHD than on PD (127.8 PTPY; 95% CI, 118.9 to 137.2 versus 93.3 PTPY; 95% CI, 91.5 to 95.1). However, HHD was associated with a slightly lower adjusted risk of cardiovascular events than PD (hazard ratio [HR], 0.92; 95% CI, 0.85 to 0.997). Compared with patients on PD, patients on HHD had 42% lower adjusted risk of stroke (HR, 0.58; 95% CI, 0.48 to 0.71), 17% lower adjusted risk of acute coronary syndrome (HR, 0.83; 95% CI, 0.72 to 0.95), and no difference in risk of heart failure (HR, 1.05; 95% CI, 0.94 to 1.16). HHD was associated with 22% lower adjusted risk of cardiovascular death (HR, 0.78; 95% CI, 0.71 to 0.86) and 8% lower adjusted risk of all-cause death (HR, 0.92; 95% CI, 0.87 to 0.97) as compared with PD.

**Conclusions:**

Relative to PD, HHD is associated with decreased risk of stroke, acute coronary syndrome, cardiovascular death, and all-cause death. Further studies are needed to better understand the factors associated with differences in cardiovascular outcomes by type of home dialysis modality in patients with kidney failure.

## Visual Abstract

**Figure:**
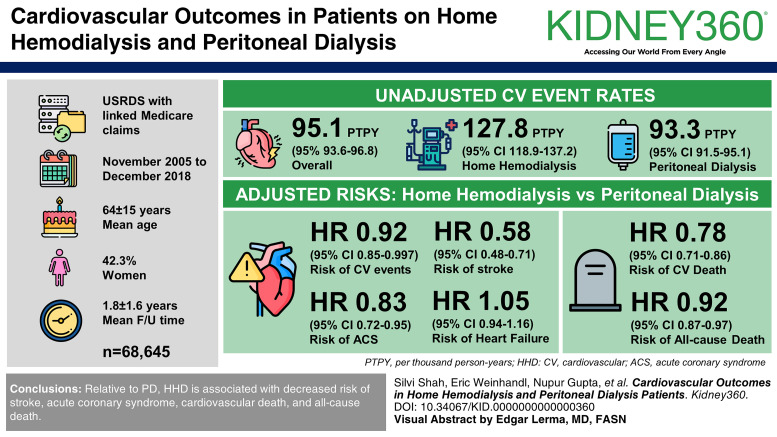

## Introduction

Cardiovascular disease remains the leading cause of morbidity and mortality among people with kidney failure. Cardiovascular disease is present in >50% of patients undergoing dialysis, and the risk of cardiovascular death in the dialysis population is 20 times higher than in the general population.^1^ This increased risk may be explained by the high prevalence of cardiovascular disease at dialysis initiation and inflammation associated with kidney failure, along with the dialysis-specific risk factors of volume overload, vascular calcification, and oxidative stress.^2^

Currently in the United States, the prevalence of home dialysis use among the population with ESKD is 13.7%, with 2.1% undergoing home hemodialysis (HHD) and 11.6% doing peritoneal dialysis (PD).^3^ A new kidney health policy in the United States, Advancing American Kidney Health Initiative, encourages patient-centered care, such as with home dialysis therapies, including both HHD and PD.^4^ Studies have demonstrated the cardiovascular benefit of frequent hemodialysis as compared with conventional three times per week hemodialysis. For example, patients receiving more frequent hemodialysis have improved BP control, lower risk of intradialytic hypotension per treatment, and regression of left ventricular mass.^5–7^ However, there is little information documenting differences in cardiovascular outcomes by home dialysis modalities.^8^ Prior reports have shown lower rates of cardiovascular hospitalizations in patients on HHD relative to PD, but did not look at individual cardiovascular (CV) events.^9,10^ Direct comparisons between the two home dialysis modalities are further complicated by small sample sizes and differences in the usual time in the natural history of ESKD that each modality is prescribed.^11,12^

Medical comorbidities, patients' preferences and lifestyle, and social characteristics and support systems may influence the selection of home dialysis modalities.^13,14^ Knowledge of comparative clinical outcomes between PD and HHD will help to inform shared decision making during modality discussion, empower wider adaptation of dialysis therapies, and lead to better interdisciplinary and equitable care. We compared cardiovascular outcomes among patients on incident HHD and PD using the United States Renal Data System (USRDS) from 2005 to 2018. Our primary aim was to compare the risks of cardiovascular events, cardiovascular death, and all-cause death in patients on daily HHD and PD.

## Methods

### Study Population and Data Sources

**Figure 1:**
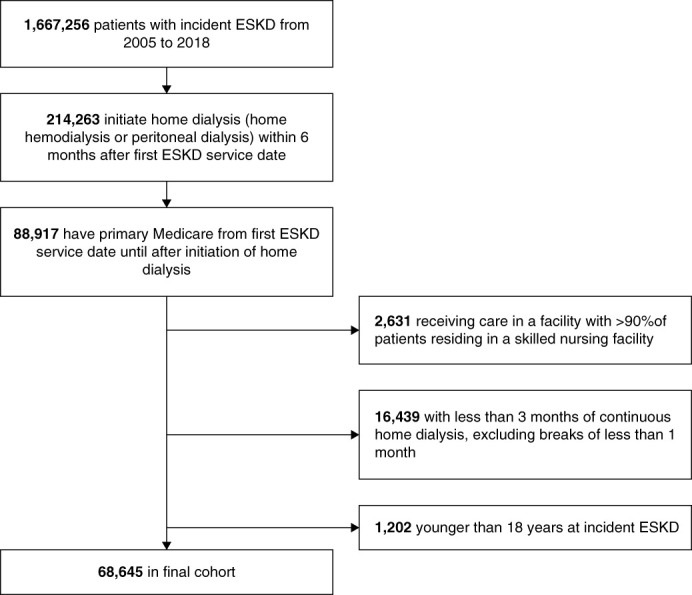
Study cohort selection flow diagram.

The study cohort included 68,645 individuals 18 years and older from USRDS with linked claims for Medicare Part A and Part B or Medicare Primary Other as the primary payer, who had incident ESKD between January 1, 2005, and December 31, 2018, and who had initiated home dialysis within 6 months of the first ESKD service date. Patients with <3 months of continuous home dialysis (excluding breaks of <1 month) and those receiving care in a facility with >90% of patients residing in a skilled nursing facility were excluded. Figure 1 shows the cohort derivation. The University of Cincinnati Institutional Review Board Committee deemed the study exempt because the data were deidentified.

### Covariates

The major predictor was type of home dialysis modality, defined as HHD or PD. Information on the covariates was obtained from the Centers for Medicare & Medicaid Services (CMS)-2728 form, USRDS patients file, USRDS treatment history file, and USRDS residence file.^15^ The USRDS patients file was used to obtain information on the date of the first ESKD service, age, sex, race/ethnicity, region, date of death, and cause of death. The USRDS treatment history file was used to identify dates and types of dialysis modalities. The USRDS residence file was used to obtain information on patients' zip codes of current residence at study entry. These zip codes were combined with zip code–level data from the United States Census Bureau American Community Survey 5-year estimates from 2007 to 2011 to determine neighborhood socioeconomic status, which we defined as the percentage of zip code residents living below the federal poverty level and grouped similarly to the United States Census Bureau literature into five categories: I (<13.8%), II (13.8%–19.9%), III (20.0%–39.9%), IV (40% or more), and unknown.^16^ The CMS form 2728, which is filed at the time of initiation of dialysis, was used to collect information on body mass index; dialysis modality; cause of kidney failure; comorbidities (cardiovascular disease, hypertension, diabetes mellitus, chronic obstructive pulmonary disease, cancer, and smoking); predialysis nephrology care; laboratory data; and poor functional status defined by inability to ambulate, inability to transfer, or need of assistance with daily activities. History of cardiovascular disease at baseline was defined by the presence of heart failure, atherosclerotic heart disease, peripheral vascular disease, amputation, or cerebrovascular accident as specified in the form CMS-2728. Patients with unavailable information on other covariates were categorized into a missing group for that covariate, as presented in Table 1.

**Table 1:** Patient characteristics by type of home dialysis modality

Patient Characteristic	All *n*=68,645	Peritoneal Dialysis *n*=63,931 (93.1%)	Home Hemodialysis *n*=4714 (6.9%)	*P* Value (Peritoneal Dialysis versus Home Hemodialysis)
**Sex**				0.245
Male	57.7	57.7	58.5	
Female	42.3	42.3	41.5	
**Race**				<0.001
Asian	3.8	3.9	2.0	
Black	18.1	17.9	21.9	
Hispanic	9.8	10.1	5.8	
Native American	0.9	1.0	0.4	
White	66.5	66.3	69.2	
Unknown/other	0.9	0.9	0.6	
**Age** ^a^	64 (15)	63 (15)	68 (13)	<0.001
				<0.001
18–29	2.7	2.8	1.1	
30–39	5.6	5.7	3.0	
40–49	9.5	9.8	5.6	
50–59	15.4	15.7	12.0	
60–69	27.7	27.7	28.7	
70–79	27.5	27.3	30.1	
≥80	11.6	11.0	19.6	
**Body mass index** ^a^	29.1 (7.1)	29 (7)	30.5 (9)	<0.001
				<0.001
<18.5	2.3	2.2	3.3	
18.5–24.99	27.7	27.9	25.7	
25–29.99	31.3	31.6	26.0	
≥30	37.6	37.2	42.6	
Missing	1.1	1.0	2.4	
History of cardiovascular disease	38.3	37.5	50.3	<0.001
Hypertension	87.6	87.9	83.1	<0.001
Diabetes	52.2	51.9	56.5	<0.001
Chronic obstructive pulmonary	7.0	6.5	14.1	<0.001
Cancer	6.7	6.4	9.9	<0.001
Poor functional status	7.3	5.7	29.1	<0.001
History of smoking	6.4	6.6	3.9	<0.001
**Albumin** ^a^	3.5 (5.3)	3.5 (5.3)	3.3 (5.6)	0.005
				<0.001
<3.5	33.4	32.9	40.5	
≥3.5	40.7	42.0	22.8	
**Hemoglobin** ^a^	10.4 (12.1)	10.4 (12.5)	9.8 (1.9)	0.003
				<0.001
<11	60.3	59.9	65.8	
11–12	15.2	15.5	10.9	
>12	10.1	10.3	7.0	
Missing	14.3	14.2	16.3	
**Cause of ESKD**				<0.001
Diabetes	44.5	44.7	41.0	
Glomerulonephritis	8.8	9.1	4.7	
Secondary glomerulonephritis/vasculitis	2.1	2.2	1.3	
Interstitial nephritis	2.7	2.7	2.7	
Hypertension/large vessel disease	30.1	29.8	34.0	
Cystic/hereditary/congenital	4.1	4.2	2.6	
Neoplasms/tumors	1.7	1.7	2.6	
Other	6.0	5.7	11.1	
**Prior nephrology care**				<0.001
None	11.7	11.5	14.2	
≤1 yr	37.8	38.4	29.3	
>1 yr	40.2	41.2	26.7	
Unknown	10.3	8.9	29.8	
**Neighborhood poverty**				<0.001
<12.8%	66.3	66.0	69.5	
12.8%–19.9%	17.5	17.7	15.4	
20%–39.9%	13.7	13.7	13.5	
≥40%	1.1	1.1	0.7	
Unknown	1.4	1.5	1.0	
**Region**				<0.001
Northeast	12.2	12.4	9.5	
South	43.0	43.6	35.7	
Midwest	26.0	24.6	45.0	
West	18.7	19.4	9.8	

Continuous variables were summarized using means and standard SDs; *t* tests were used to test for differences in means.

a Reported in mean (SD).

Patients receiving care in a facility with >90% of patients residing in a skilled nursing facility were excluded. During the study era, an increasing but nonetheless small share of patients dialyzing at home, as indicated in USRDS data, resided in a skilled nursing facility, not a private residence. This trend has been more pronounced with HHD than with PD because of state regulations that have permitted the construction of dialysis dens. USRDS data do not clearly indicate whether a patient resides in a private residence or skilled nursing facility, and data also fail to indicate whether a Medicare-certified dialysis facility actually provides on-site hemodialysis to skilled nursing facilities. To address this issue in an objective manner that was applicable to both dialytic modalities, we identified first-time nursing home residency after home dialysis initiation by identifying the date of the first physician claim with place of service code 31 (“skilled nursing facility”) or 32 (“nursing facility”) or *Current Procedural Terminology* codes 99304–99318, indicating evaluation and management services in a nursing facility.^17^ We parameterized nursing home residency as a monotonic binary factor equal to 0 from home dialysis initiation until the date of the first qualifying claim and equal to 1 thereafter. This approach is supported by Chen *et al.*, who showed that even a short stay in a nursing facility was associated with markedly higher risks of death and hospitalization during 1 year of follow-up.^18^

### Outcomes

The *primary outcomes* were the occurrence of a composite cardiovascular event, defined as hospitalization with a primary diagnosis of acute coronary syndrome (myocardial infarction or unstable angina), heart failure, or stroke; separate occurrence of acute coronary syndrome, heart failure, or stroke; cardiovascular death; and all-cause death.^19^ Information on cardiovascular events was obtained from the Medicare claims data using the International Classification of Disease, Clinical Modification (ICD-9-CM/ICD-10-CM) diagnostic codes.^20–22^ Myocardial infarction, unstable angina, heart failure, and stroke were identified by ICD-9-CM codes as presented in Supplemental Material 1.^23–25^ Death dates and causes of death as reported on CMS form 2746 were obtained from the USRDS patients file. Cardiovascular death indicated that the reported code for the cause of death was a cardiovascular one and included myocardial infarction; acute pericarditis, including cardiac tamponade; atherosclerotic heart disease; cardiomyopathy; cardiac arrhythmia; cardiac arrest cause unknown; valvular heart disease; congestive heart failure; cerebrovascular accident, including intracranial hemorrhage; and ischemic brain damage/anoxic encephalopathy.

### Statistical Analyses

Descriptive statistics were used to describe patients' characteristics at dialysis initiation. Categorical variables were summarized using frequencies and percentages, with chi-square or Fisher exact tests used to test for differences between HHD and PD. Continuous variables were summarized using means and SDs, with *t* tests used to test for differences in means. Rates were expressed as the number of events per thousand person-years (PTPY); exact Poisson confidence intervals (CIs) were calculated, and significance was tested using unadjusted Poisson models.

To test the association of type of home dialysis modality with outcomes, adjusted for our covariates, multivariable Cox proportional hazards time-to-event models were constructed. All observation times were censored at the end of an initial home dialysis period, which was continuous, except for breaks of less than one month, end of primary Medicare coverage, or end of the study period—December 31st, 2018; thus, death, recovery, and transplant were implicitly censored as well. Nursing home was used as a time-dependent covariate. The risk estimates were expressed as hazard ratios (HRs) and their 95% CIs. We adjusted directly for covariates in the Cox regression analyses rather than using propensity score matching using those covariates to see the separate effects of covariates on the outcomes. Because residual confounding does not go away with propensity score matching, some statistical experts have criticized matching because it discards information from some unexposed patients, resulting in less precise estimates of the association between exposure and outcome.

All statistical analyses were conducted using SAS version 9.4, with two-sided hypothesis testing and a *P* value of <0.05 as the criterion for statistical significance.

## Results

### Baseline Characteristics

Patients' mean age at study entry was 64±15 years, and 42.3% were women. Regarding race/ethnicity, 66.5% were non-Hispanic White, 18.1% were non-Hispanic Black, 9.8% were Hispanic, 3.8% were Asian, and 0.9% were Native American. Only 11.7% did not receive predialysis nephrology care. Most of the study cohort had a history of hypertension (87.6%) and 38.3% had a history of cardiovascular disease. Diabetes (44.5%) was the most common attributed cause of kidney failure among patients on dialysis, followed by hypertension (30.1%). Table 1 summarizes the baseline characteristics of the study cohort.

The mean age at study entry was higher for patients on HHD than for patients on PD (68±13 versus 63±15 years). The majority of both patients on HHD and PD were of White race/ethnicity. Patients on HHD were less likely to be Hispanic (5.8% versus 10.1%). Patients on HHD were more likely to have comorbidities of cardiovascular disease (50.3% versus 37.5%), diabetes (56.5% versus 51.9%), and poor functional status (29.1% versus 5.7%), Patients on HHD were more likely to have kidney failure due to hypertension/large vessel disease (34.0% versus 29.8%) and less likely to have kidney failure due to glomerulonephritis (4.7% versus 9.1%). More patients on HHD resided in the mid-Western United States region (45.0% versus 24.6%). More patients on HHD lived in zip codes of higher socioeconomic status (<13.8% poverty category) (69.5% versus 66.0%). Patients on HHD had more unfavorable laboratory parameters of albumin <3.5 mg/dl (40.5% versus 32.9%) and hemoglobin <11 g/dl (65.8% versus 59.9%). Table 1 summarizes the characteristics of patients by type of home dialysis modality.

### Outcomes

#### Cardiovascular Events

The cardiovascular event rate was 95.1 PTPY (95% CI, 93.3 to 96.8), the most common of which was heart failure (44.4 PTPY, 95% CI, 43.2 to 45.6). The mean time of follow-up was 1.8±1.6 years overall, 1.4±1.6 years for patients on HHD, and 1.9±1.6 years for patients on PD. Overall, the cardiovascular event rate was higher in patients on HHD (127.8 PTPY; 95% CI, 118.9 to 137.2), as compared with those on PD (93.3 PTPY; 95% CI, 91.5 to 95.1). Rates of heart failure (76.5 PTPY; 95% CI, 69.9 to 83.7 versus 42.6 PTPY; 95% CI, 41.4 to 43.8) were higher in patients on HHD than in those on PD. The stroke event rate was slightly lower in patients on HHD (19.7 PTPY; 95% CI, 16.4 to 23.4), as compared with those on PD (23.6 PTPY; 95% CI, 22.7 to 24.5). Rates of acute coronary syndrome did not differ significantly between patients on HHD and PD (35.1 PTPY; 95% CI, 30.7 to 39.9 versus 33.4 PTPY; 95% CI, 32.4 to 34.5). Table 2 provides the unadjusted cardiovascular rates by type of dialysis modality (events, events per 1000 person-years).

**Table 2:** Rates of cardiovascular events, all-cause death, and cardiovascular death per thousand person-years by type of home dialysis modality

Variable	Overall	Home Hemodialysis	Peritoneal Dialysis	*P* Values (Peritoneal Dialysis versus Home Hemodialysis)
Cardiovascular events	95.1 (93.3–96.8)	127.8 (118.9–137.2)	93.3 (91.5–95.1)	<0.001
Heart failure	44.4 (43.2–45.6)	76.5 (69.9–83.7)	42.6 (41.4–43.8)	<0.001
Stroke	23.4 (22.5–24.3)	19.7 (16.4–23.4)	23.6 (22.7–24.5)	<0.001
Acute coronary syndrome	33.5 (32.5–34.6)	35.1 (30.7–39.9)	33.4 (32.4–34.5)	0.394
Cardiovascular death	58.8 (57.5–60.1)	85.7 (78.8–93.0)	57.3 (55.9–58.6)	<0.001
All-cause death	135.5 (133.5–137.6)	255.6 (243.7–268.0)	128.8 (126.7–130.8)	<0.001

Rates were expressed as the number of events per thousand person-years (PTPY); exact Poisson confidence intervals were calculated; significance was tested using unadjusted Poisson models.

**Figure 2:**
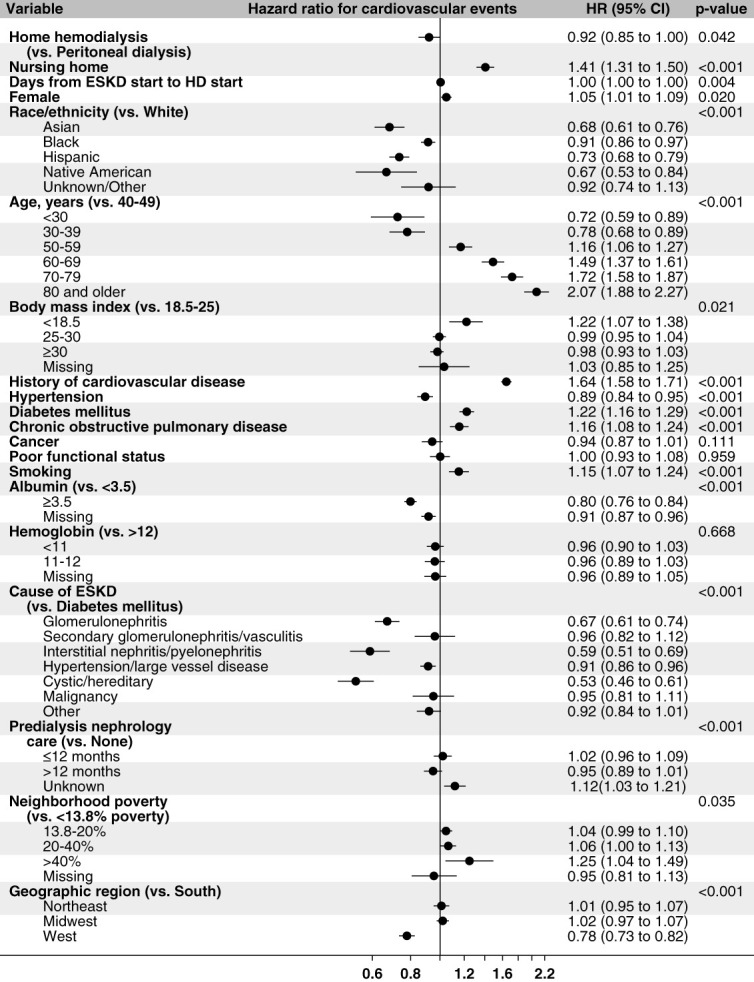
**Time-to-event model showing adjusted hazard ratios for cardiovascular events.** *The model predicts time to cardiovascular events, with patients censored at the time of end of continuous home dialysis (other than breaks of <30 days), end of time with primary Medicare, or end of the study period; nursing home stay is a time-dependent covariate.

**Figure 3:**
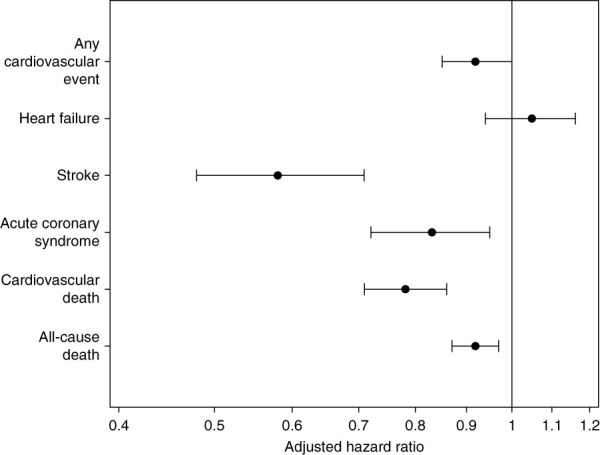
**Time-to-event model showing adjusted hazard ratios for cardiovascular death.** *The model predicts time to cardiovascular death, with patients censored at the time of end of continuous home dialysis (other than breaks of <30 days), end of time with primary Medicare, or end of the study period; nursing home stay is a time-dependent covariate.

In the regression models adjusted for demographics, nursing home residency, socioeconomic characteristics, and other clinical characteristics**,** the risk of cardiovascular events was slightly lower in patients on HHD than in patients on PD (HR, 0.92; 95% CI, 0.85 to 0.997) (Figure 2). Patients on HHD experienced a lower risk of stroke (HR, 0.58; 95% CI, 0.48 to 0.71) and a lower risk of acute coronary syndrome (HR, 0.83; 95% CI, 0.72 to 0.95), as compared with those on PD. The risk of heart failure was similar in both modalities (HR, 1.05; 95% CI, 0.94 to 1.16) (Figure 3).

#### Cardiovascular Death

**Figure 4:**
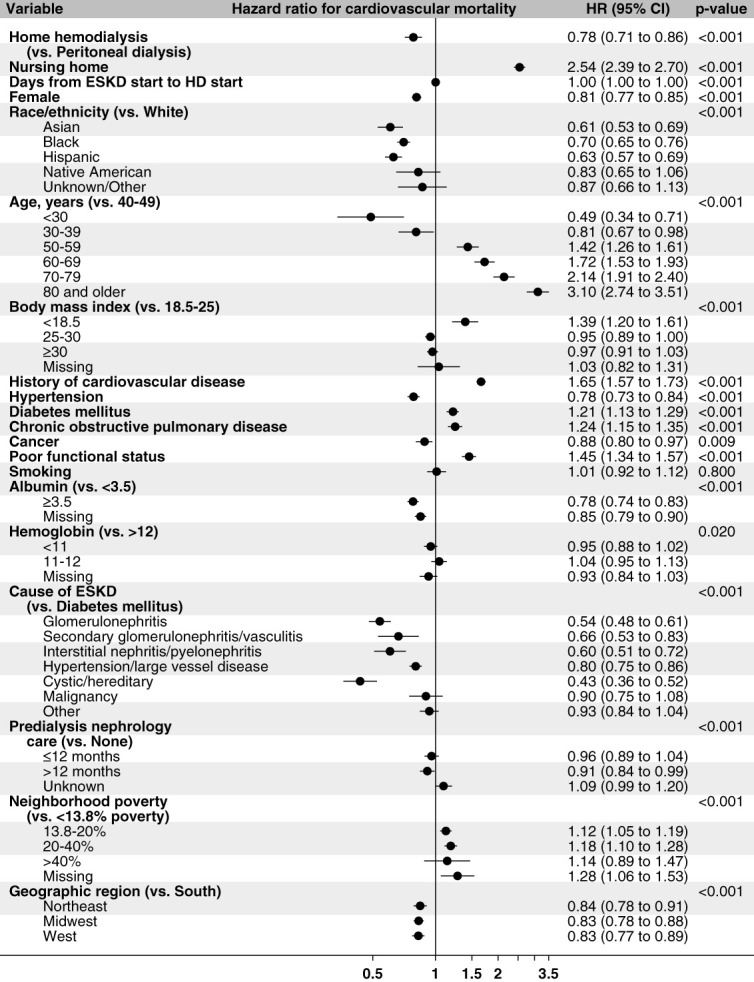
**Time-to-event model hazard ratios for home hemodialysis versus peritoneal dialysis for outcomes of cardiovascular events, heart failure, stroke, acute coronary syndrome, cardiovascular death, and all-cause death.** *The models predict time to cardiovascular events, heart failure, stroke, and acute coronary syndrome, cardiovascular death and all-cause death censored for end of continuous home dialysis (other than breaks of <30 days), end of primary Medicare coverage, and end of the study period. Hazard ratios are adjusted for covariates of type of home dialysis, age, sex, race/ethnicity, body mass index, history of cardiovascular disease, hypertension, diabetes mellitus, chronic obstructive pulmonary disease, cancer, smoking, functional status, albumin, hemoglobin, cause of ESKD, predialysis nephrology care, neighborhood poverty, geographic region, nursing home stay, and time from start of ESKD to the start of home dialysis; nursing home stay is a time-dependent covariate.

In the incident home dialysis population, the event rate for cardiovascular death was 58.8 PTPY (95% CI, 57.5 to 60.1). The event rate for cardiovascular death was higher in HHD (85.7 PTPY; 95% CI, 78.8 to 93.0), as compared with PD (57.3 PTPY; 95% CI, 55.9 to 58.6) (Table 2). In the adjusted time-to-event model predicting cardiovascular death, HHD had a lower risk of cardiovascular death (HR, 0.78; 95% CI, 0.71 to 0.86) than PD (Figure 4).

#### All-Cause Death

In the incident home dialysis population, the event rate for all cause-death was 135.5 PTPY (95% CI, 133.5 to 137.6). The event rate for all-cause death was higher in HHD (255.6 PTPY; 95% CI, 243.7 to 268.0), as compared with PD (128.8 PTPY; 95% CI, 126.7 to 130.8) (Table 2). In the adjusted time-to-event model predicting all-cause death, HHD had a lower risk of all-cause death (HR, 0.92; 95% CI, 0.87 to 0.97) than PD (Figure 3).

## Discussion

Our study examined the cardiovascular outcomes among patients on incident PD and HHD from 2005 to 2018 in the United States. We found that patients on incident HHD had a lower adjusted risk of cardiovascular events, cardiovascular death, and all-cause death. Further classifying the cardiovascular events, the risk of heart failure was similar, although patients on HHD had a lower risk of strokes and a lower risk of acute coronary syndrome than patients on PD.

Our study shows a higher unadjusted risk but a slightly lower adjusted risk of cardiovascular events in HHD relative to PD; the differences between the two estimates are likely because of differences in baseline characteristics between the two home dialysis modality groups. For example, patients on HHD were older and had more diabetes than patients on PD, but after adjustment with these covariates, patients on home dialysis had lower hazards of cardiovascular events than those on PD. Most patients on HHD in the United States are prescribed at least four treatments per week, and a subgroup are prescribed nocturnal hemodialysis, which typically involves >20 treatment hours per week. Cardiovascular benefit of intensive hemodialysis as compared with thrice-weekly in-center dialysis has been reported because of its association with reductions in left ventricular mass and BP, decreased use of antihypertensive medications, and increased left ventricular ejection fraction. While no studies have specifically compared incidence of CV events in patients on incident HHD and PD, Weinhandl *et al.* showed a 11% lower risk of cardiovascular hospitalization and a shorter length of stay for cardiovascular hospitalization in matched patients on HHD as compared with patients on PD.^9^ Similarly, Suri *et al.* reported a lower risk of cardiovascular hospitalizations in HHD relative to PD.^10^ PD may negatively alter the risk of cardiovascular morbidity. Fluid overload is frequently seen in patients on PD because of poor adherence to fluid intake restriction, low effluent drain volume, high transporter status, infrequent prescription changes, mechanical complications of PD catheters, or loss of residual kidney function.^26,27^ Patients on HHD have a lower adjusted risk of CV mortality as compared with patients on PD.

Nocturnal HHD stabilizes coronary calcification burden and improves endothelial vasodilation, baroreflex sensitivity, and arterial compliance.^28,29^ CKD–mineral and bone disorder, including high levels of calcium, phosphorus, alkaline phosphatase, and parathyroid hormone, results in vascular calcification and remains an important contributor to cardiovascular mortality.^30,31^ Enhanced solute clearance coupled with volume removal with more frequent or longer HHD results in lower cardiovascular mortality because of improved control of BP, improved control of parameters associated with bone and mineral metabolism, reductions in ventricular volumes, and regression of left ventricular mass.^5,32^ Oxidative stress from advanced glycation end products formed^28^ during heat sterilization of dialysate has also emerged in the recent decades as a novel nontraditional, uremia-related risk factor of increased cardiovascular mortality and morbidity in patients on PD.^33^

HHD was associated with slightly better adjusted survival as compared with PD. The Australia and New Zealand Dialysis and Transplant Registry study reported a much higher improvement in survival, with 53% reduction in mortality in 706 patients on patients compared with 10,710 patients on PD.^11^ However, this study was limited by the sole inclusion of patients started very early on home dialysis (within 90 days), which, especially for HHD, might have introduced a selection bias. In addition, the greater association of HHD with lower mortality may also involve stricter selection criteria favoring healthier patients for HHD in these centers less experienced with HHD therapy. A Canadian study showed a 34% lower risk of mortality for patients on incident HHD compared with those on incident PD. The risks of mortality with HHD were more pronounced for older cohorts from 2005 to 2019 and were not significantly different from 2011 to 2013.^12^ These findings may be possibly related to the inclusion of higher acuity patients in HHD programs over time and simultaneous improvement in the PD technique and patient survival. By contrast, a study from the United States demonstrated that mortality rates in 4201 prevalent patients on HHD and matched patients on PD were not statistically significant when the analysis was restricted to patients who started home dialysis within 6 months of kidney replacement therapy initiation.^9^ While we also restricted analysis to patients who started home dialysis within 6 months of onset of kidney failure, these differences could be attributed to the variation in prescription patterns and practices (short/daily versus longer/nocturnal). Higher risk of mortality in patients on PD may also be explained by higher risk infection–related hospitalizations and morbidity.^34,35^ As reported in other home dialysis studies and represented in the baseline characteristics of this cohort, patients selecting PD and HHD are traditionally very different in terms of demographics and comorbidity burden, and statistical strategies may not always address these differences entirely.^9–11^

Our study has some limitations. First, the observational design precludes determination of causality. Second, comorbidity reporting on the Medical Evidence Report form has not been validated, and prior studies have shown underreporting of comorbid conditions that may have resulted in some nondifferential misclassification. Third, the details on treatment sessions and prescription patterns are not available in Medicare. Fourth, there is variability in the quality and completeness of the data recorded on form 2728 of the USRDS data. Fifth, we did not have data for residual renal function and urine output for patients on home dialysis, which remains a limitation of the study. Our study has the following strengths. First, because we used the largest registry of patients on dialysis in the United States and restricted to patients with primary Medicare, it captures all patients with ESKD and all hospitalizations, therefore, accurately predicting the rates of cardiovascular events and cardiovascular deaths in both HHD and PD for this high-risk population. Second, it included 68,645 patients, with the largest sample size for the home dialysis population in the literature so far.

In conclusion, HHD relative to PD is associated with lower adjusted risks of cardiovascular events, including those of stroke and acute coronary syndrome; lower adjusted risks of cardiovascular mortality; and lower adjusted risk of all-cause mortality.

## Supplementary Material

SUPPLEMENTARY MATERIAL

## Data Availability

All data is included in the manuscript and/or supporting information.

## Supplemental Material

Supplemental Material 1. Discharge diagnoses indicative of cardiovascular events.

## References

[1] Foley RN Parfrey PS Sarnak MJ. Clinical epidemiology of cardiovascular disease in chronic renal disease. Am J Kidney Dis. 1998;32(5 suppl 3):S112–S119. doi:10.1053/ajkd.1998.v32.pm9820470 9820470

[2] Sarnak MJ Amann K Bangalore S, . Chronic kidney disease and coronary artery disease: JACC state-of-the-art review. J Am Coll Cardiol. 2019;74(14):1823–1838. doi:10.1016/j.jacc.2019.08.1017 31582143

[3] Johansen KL Chertow GM Gilbertson DT, . US renal data system 2021 annual data report: epidemiology of kidney disease in the United States. Am J Kidney Dis. 2022;79(4 suppl 1):A8–A12. doi:10.1053/j.ajkd.2022.02.001 35331382 PMC8935019

[4] Advancing American Kidney Health. Accessed March 22, 2022. https://aspe.hhs.gov/sites/default/files/private/pdf/262046/AdvancingAmericanKidneyHealth.pdf

[5] Chertow GM Levin NW Beck GJ, . In-center hemodialysis six times per week versus three times per week. N Engl J Med. 2010;363(24):2287–2300. doi:10.1056/NEJMoa1001593 21091062 PMC3042140

[6] Chan CT Greene T Chertow GM, . Determinants of left ventricular mass in patients on hemodialysis: frequent Hemodialysis Network (FHN) Trials. Circ Cardiovasc Imaging. 2012;5(2):251–261. doi:10.1161/CIRCIMAGING.111.969923 22360996 PMC3328963

[7] Chan C Floras JS Miller JA Pierratos A. Improvement in ejection fraction by nocturnal haemodialysis in end-stage renal failure patients with coexisting heart failure. Nephrol Dial Transplant. 2002;17(8):1518–1521. doi:10.1093/ndt/17.8.1518 12147805

[8] Sarnak MJ Auguste BL Brown E, . Cardiovascular effects of home dialysis therapies: a scientific statement from the American Heart Association. Circulation. 2022;146(11):e146–e164. doi:10.1161/cir.0000000000001088 35968722

[9] Weinhandl ED Gilbertson DT Collins AJ. Mortality, hospitalization, and technique failure in daily home hemodialysis and matched peritoneal dialysis patients: a matched cohort study. Am J Kidney Dis. 2016;67(1):98–110. doi:10.1053/j.ajkd.2015.07.014 26319755

[10] Suri RS Li L Nesrallah GE. The risk of hospitalization and modality failure with home dialysis. Kidney Int. 2015;88(2):360–368. doi:10.1038/ki.2015.68 25786099 PMC4526768

[11] Nadeau-Fredette AC Hawley CM Pascoe EM, . An incident cohort study comparing survival on home hemodialysis and peritoneal dialysis (Australia and New Zealand dialysis and transplantation registry). Clin J Am Soc Nephrol. 2015;10(8):1397–1407. doi:10.2215/CJN.00840115 26068181 PMC4527016

[12] Nadeau-Fredette AC Tennankore KK Perl J Bargman JM Johnson DW Chan CT. Home hemodialysis and peritoneal dialysis patient and technique survival in Canada. Kidney Int Rep. 2020;5(11):1965–1973. doi:10.1016/j.ekir.2020.08.020 33163717 PMC7609902

[13] Whittaker AA Albee BJ. Factors influencing patient selection of dialysis treatment modality. ANNA J. 1996;23(4):369–377; discussion 376-377. PMID: 8900682. 8900682

[14] de Jong RW Stel VS Rahmel A, . Patient-reported factors influencing the choice of their kidney replacement treatment modality. Nephrol Dial Transplant. 2022;37(3):477–488. doi:10.1093/ndt/gfab059 33677544 PMC8875472

[15] U.S. Renal Data System. 2015 Researcher’s Guide to the USRDS Database. National Institutes of Health, National Institute of Diabetes and Digestive and Kidney Diseases; 2015.

[16] Johns TS Estrella MM Crews DC, . Neighborhood socioeconomic status, race, and mortality in young adult dialysis patients. J Am Soc Nephrol. 2014;25(11):2649–2657. doi:10.1681/ASN.2013111207 24925723 PMC4214533

[17] Weinhandl ED Liu J Gilbertson DT Wetmore JB Johansen KL. Associations of COVID-19 outcomes with dialysis modalities and settings. Clin J Am Soc Nephrol. 2022;17(10):1526–1534. doi:10.2215/CJN.03400322 36400565 PMC9528267

[18] Chen S Slowey M Ashby VB, . Nursing home status adjustment for standardized mortality and hospitalization in dialysis facility reports. Kidney Med. 2023;5(2):100580. doi:10.1016/j.xkme.2022.100580 36712314 PMC9879984

[19] Johnson DW Dent H Hawley CM, . Association of dialysis modality and cardiovascular mortality in incident dialysis patients. Clin J Am Soc Nephrol. 2009;4(10):1620–1628. doi:10.2215/CJN.01750309 19729428 PMC2758255

[20] Go AS Chertow GM Fan D McCulloch CE Hsu CY. Chronic kidney disease and the risks of death, cardiovascular events, and hospitalization. N Engl J Med. 2004;351(13):1296–1305. doi:10.1056/NEJMoa041031 15385656

[21] Nakayama M Sato T Miyazaki M, . Increased risk of cardiovascular events and mortality among non-diabetic chronic kidney disease patients with hypertensive nephropathy: the Gonryo study. Hypertens Res. 2011;34(10):1106–1110. doi:10.1038/hr.2011.96 21796127

[22] Diagnosis Code Set General Equivalence Mappings. Accessed March 23, 2023. https://www.cms.gov/files/document/diagnosis-code-set-general-equivalence-mappings-icd-10-cm-icd-9-cm-and-icd-9-cm-icd-10-cm.pdf

[23] Nadkarni GN Patel AA Konstantinidis I, . Dialysis requiring acute kidney injury in acute cerebrovascular accident hospitalizations. Stroke. 2015;46(11):3226–3231. doi:10.1161/strokeaha.115.010985 26486869

[24] Thakar CV Parikh PJ Liu Y. Acute kidney injury (AKI) and risk of readmissions in patients with heart failure. Am J Cardiol. 2012;109(10):1482–1486. doi:10.1016/j.amjcard.2012.01.362 22381163

[25] Quan H Parsons GA Ghali WA. Validity of information on comorbidity derived rom ICD-9-CCM administrative data. Med Care. 2002;40(8):675–685. doi:10.1097/00005650-200208000-00007 12187181

[26] Van Biesen W Williams JD Covic AC, . Fluid status in peritoneal dialysis patients: the European Body Composition Monitoring (EuroBCM) study cohort. PLoS One. 2011;6(2):e17148. doi:10.1371/journal.pone.0017148 21390320 PMC3044747

[27] Auguste BL Bargman JM. Peritoneal dialysis prescription and adequacy in clinical practice: core curriculum 2023. Am J Kidney Dis. 2023;81(1):100–109. doi:10.1053/j.ajkd.2022.07.004 36208963

[28] Yuen D Pierratos A Richardson RM Chan CT. The natural history of coronary calcification progression in a cohort of nocturnal haemodialysis patients. Nephrol Dial Transplant. 2006;21(5):1407–1412. doi:10.1093/ndt/gfl021 16504981

[29] Chan CT Jain V Picton P Pierratos A Floras JS. Nocturnal hemodialysis increases arterial baroreflex sensitivity and compliance and normalizes blood pressure of hypertensive patients with end-stage renal disease. Kidney Int. 2005;68(1):338–344. doi:10.1111/j.1523-1755.2005.00411.x 15954925

[30] Tentori F Blayney MJ Albert JM, . Mortality risk for dialysis patients with different levels of serum calcium, phosphorus, and PTH: the Dialysis Outcomes and Practice Patterns Study (DOPPS). Am J Kidney Dis. 2008;52(3):519–530. doi:10.1053/j.ajkd.2008.03.020 18514987

[31] Stevens LA Djurdjev O Cardew S Cameron E Levin A. Calcium, phosphate, and parathyroid hormone levels in combination and as a function of dialysis duration predict mortality: evidence for the complexity of the association between mineral metabolism and outcomes. J Am Soc Nephrol. 2004;15(3):770–779. doi:10.1097/01.ASN.0000113243.24155.2f 14978180

[32] Culleton BF Walsh M Klarenbach SW, . Effect of frequent nocturnal hemodialysis vs conventional hemodialysis on left ventricular mass and quality of life: a randomized controlled trial. JAMA. 2007;298(11):1291–1299. doi:10.1001/jama.298.11.1291 17878421

[33] Roumeliotis S Dounousi E Salmas M Eleftheriadis T Liakopoulos V. Unfavorable effects of peritoneal dialysis solutions on the peritoneal membrane: the role of oxidative stress. Biomolecules. 2020;10(5):768. doi:10.3390/biom10050768 32423139 PMC7277773

[34] Laurin LP Harrak H Elftouh N Ouimet D Vallée M Lafrance JP. Outcomes of infection-related hospitalization according to dialysis modality. Clin J Am Soc Nephrol. 2015;10(5):817–824. doi:10.2215/CJN.09210914 25818336 PMC4422244

[35] Perl J Fuller DS Bieber BA, . Peritoneal dialysis-related infection rates and outcomes: results from the peritoneal dialysis outcomes and practice patterns study (PDOPPS). Am J Kidney Dis. 2020;76(1):42–53. doi:10.1053/j.ajkd.2019.09.016 31932094

