# Supplementary material for: Cardiovascular Outcomes in Patients on Home Hemodialysis and Peritoneal Dialysis
Source: Kidney360. 2024 Feb 1;5(2):205–15. doi: 10.34067/KID.0000000000000360 (PMC10914201; doi:10.34067/KID.0000000000000360)
Supplement: SUPPLEMENTARY MATERIAL [file kidney360-5-205-s001.pdf]

Supplemental Material 1. Discharge diagnoses indicative of cardiovascular events.

Event	ICD-9 diagnosis codes
Heart failure	402.01, 402.11, 402.91, 404.01, 404.03, 404.11, 404.13, 404.91, 404.93, 428.0, 428.1, 428.20, 428.21, 428.22, 428.23, 428.30, 428.31, 428.32, 428.33, 428.40, 428.41, 428.42, 428.43, 428.9
Stroke	433.00, 433.01, 433.10, 433.11, 433.20, 433.21, 433.30, 433.31, 433.80, 433.81, 433.90, 433.91, 434.00, 434.01, 434.10, 434.11, 434.90, 434.91, 436, 437.1
Acute coronary syndrome	410.01, 410.01, 410.02, 410.10, 410.11, 410.12, 410.30, 410.31, 410.32, 410.40, 410.41, 410.42, 410.50, 410.51, 410.52, 410.60, 410.61, 410.62, 410.70, 410.71, 410.72, 410.80, 410.81, 410.82, 410.90, 410.91, 410.92, 411.0, 411.1, 411.81, 411.89
